# Volumetric modulated arc therapy based total marrow and lymphoid irradiation: Workflow and clinical experience

**DOI:** 10.3389/fonc.2022.1042652

**Published:** 2023-01-05

**Authors:** Colton Ladbury, Chunhui Han, An Liu, Jeffrey Y. C. Wong

**Affiliations:** Department of Radiation Oncology, City of Hope National Medical Center, Duarte, CA, United States

**Keywords:** TMLI (total marrow and lymphatic irradiation), IMRT (intensity modulated radiation therapy), VMAT (volumetric modulated arc therapy), radiation, dosimetry, HCT (hematopoietic cell transplant)

## Abstract

**Background:**

The aim of this study is to report historical treatment planning experience at our institution for patients receiving total marrow and lymphatic irradiation (TMLI) using volumetric modulated arc therapy (VMAT) as part of the conditioning regimen prior to hematopoietic stem cell transplant.

**Methods:**

We identified a total of fifteen patients with VMAT TMLI, ten with a prescription dose of 20 Gy (targeting the skeletal bones, lymph nodes, spleen, and spinal canal, with 12 Gy to the brain and liver) and five with a prescription dose of 12-16 Gy (targeting the skeletal bones, lymph nodes, spleen, and spinal canal). Representative dosimetric parameters including total treatment time, mean and median dose, D80, and D10 (dose covering 80% and 10% of the structure volume, respectively) for targets and normal organs were extracted and compared to historical patients treated with helical tomotherapy.

**Results:**

The median treatment time for the first and subsequent fractions was 1.5 and 1.1 hours, respectively. All the target volumes had a mean dose greater than the prescribed dose except the ribs, which had an average mean dose of 19.5 Gy. The skeletal bones had an average mean dose of 21.1 Gy. The brain and liver have average mean doses of 14.8 and 14.1 Gy, respectively. The mean lung dose had an average of 7.6 ± 0.6 Gy for the 20-Gy cohort. Relative to the prescription dose of 20 Gy, the average mean dose for the normal organ volumes ranged from 16.5% to 72.0%, and the average median dose for the normal organs ranged from 16.5% to 71.0%. Dosimetry for patients treated to 12-16 Gy fell within expected ranges based on historical helical tomotherapy plans.

**Conclusions:**

Dosimetric data in the VMAT TMLI plans at our institution are summarized for 20 Gy and 12-16 Gy cohorts. Dose distributions and treatment times are overall similar to plans generated with helical tomotherapy. TMLI may be delivered effectively using a VMAT technique, even at escalated doses.

## 1 Introduction

Total body irradiation (TBI) is a critical component of the conditioning regimen for hematopoietic cell transplantation, increasing the probability of a successful transplant by helping eradicate cancerous cells and/or decreasing risk of graft rejection (1, 2). Traditionally, TBI has been administered using two-dimensional treatment planning with an anteroposterior (AP) and posteroanterior (PA) fields (3). When myeloablative doses, typically in the range of 12-13.2 Gy, are administered, this requires shielding of critical organs such as the lungs to reduce the risk of morbidity. Organs that are not shielded receive the full prescription dose. As a result, TBI is associated with a multitude of acute and chronic complications including pneumonitis, renal dysfunction, and hypothyroidism (4). The morbidity associated with TBI treatment has proven prohibitive for achieving dose escalation, which might otherwise be a valuable means of decreasing risk of relapse (5–8).

Advances in radiation technology have offered an alternative to conventional TBI that can help overcome those shortcomings. Intensity modulated radiation therapy (IMRT), which has become widely available in the early 2000s, has the capability to provide focused and conformal dose distributions that can better target regions of interest while limiting dose to organs at risk (OARs) (9). This led to the development of total marrow and lymphatic irradiation (TMLI), which focused radiation on structures critical for reducing relapse rates (bone marrow ± lymph nodes), and sparing other organs such as the brain, lungs, heart, kidneys, and testis (10–13). This approach has been shown to reduce toxicities (14). Further, by limiting dose to OARs, dose escalation has been facilitated without excessive toxicity (15, 16).

Historically, TMLI treatments have been administered using helical tomotherapy (HT) machines due to their ability to treat the length of the body without requiring multiple isocenters and treatment fields, and therefore multiple image acquisitions for image guidance (13). To date, our institution has treated over 400 patients using HT-based TMLI. However, conventional C-arm linear accelerators are more prevalent than HT machines, and therefore a TMLI technique provides access for more patients receiving TMLI treatments. Starting in 2021, our institution began administering clinical TMLI treatments using VMAT fields. We have delivered TMLI treatments using VMAT fields on conventional linear accelerators for 15 patients with prescription dose ranging from 12 Gy to 20 Gy. Herein, we report our treatment planning and delivery experience.

## 2 Materials and methods

### 2.1 VMAT TMLI technique

#### 2.1.1 Simulation

Patients are immobilized using a thermoplastic mask from the head to shoulder region and covering the feet, in addition to a full body vacuum bag (VakLok). For patients shorter than 105-135 cm, the CT simulation scan spans the top of the skull to the bottom of the feet, in a feet-first supine position. For taller patients, two separate simulation CTs are acquired: one for the upper body in a head-first supine position and one for the lower body in a head-first supine position, with overlap in the pelvis and proximal thigh regions. Both arms are kept straight and close to the body with hands forming loose fists. Three radiopaque triangulation markers are placed in the abdominal area in the same axial plane to mark the origin of the coordinates used in the CT images. Additionally, two radiopaque markers are placed in an axial plane at the upper thigh level to assist with setup of treatment fields for the upper body and lower extremities. Lastly, a set of three radiopaque triangulation markers are placed at the mid-shin level to mark the origin for the lower-extremity CT simulation.

Computed tomography (CT) simulation is obtained using 7.5 mm slice thickness. Images are acquired with patients breathing using shallow respirations. To fully model respiratory movement, end of expiration and end of inspiration breath hold CT scans are also acquired for the thoracic and abdominal regions. CT simulation scans are then sent to the Eclipse treatment planning system (Varian Medical Systems, Palo Alto, CA). The scans are registered based on bony anatomy to generate a whole-body image set used for a single treatment plan.

To facilitate treatment planning, the upper body and lower extremity CT simulation are concatenated to form a whole-body CT image set. A commercial software application (Velocity, Varian Medical Systems, Inc., Palo Alto, California) is used to concatenate the CT images based on deformable image registration results.

#### 2.1.2 Treatment planning

All contouring and planning were performed using the Eclipse (Varian Medical Systems, Palo Alto, CA) v16.1 treatment planning system. At our institution, all structures are delineated the same way in VMAT and HT cases and the same dosimetric guidelines are used for plan optimization, facilitating comparison of the two techniques (17). Following CT simulation, normal organs and target structures are delineated on the image set according to the specific treatment protocol. Artificial intelligence based auto-segmentation algorithms are used to help contour both targets and normal organs, which are manually adjusted by the treating physician and dosimetrist as needed. Target volumes at minimum include all bones and associated marrow, major lymph node chains, and the spleen. Depending on the protocol, the brain, liver, and testes are sometimes included as target volumes. The planning target volume (PTV) includes a 5-10 mm margin on bone, cropped away from skin, esophagus, and kidney by at least 5 mm. The mandible is excluded to facilitate organ sparing. No anterior margin is used for vertebra and pelvic bones, and no inner margin us used for ribs and skull to facilitate organ sparing. Avoidance structures include the brain, eyes, lenses, optic nerves, parotid glands, oral cavity, thyroid, lungs, heart, esophagus, breasts (in females), stomach, small intestine, liver, kidneys, bladder, rectum, ovaries and uterus (in females), and testes (in males, unless included in target volume). Using the end-expiration and end-inspiration scans, respiratory motion is accounted for in relevant organs including esophagus, kidneys, spleen, and liver.

Following delineation of treatment and avoidance structures, treatment plans are generated for a Varian TrueBeam linear accelerator with a 120-leaf multi-leaf collimator (MLC), with a leaf width of 5 mm for the central 40 leaf pairs and a leaf width of 1 cm for the peripheral 20 leaf pairs. The maximum field dimension is 40 cm × 40 cm and the maximum MLC travel is 15 cm. For adult patients, four to five isocenters are typically required for the upper body to mid-thigh TMLI treatment plan, with two VMAT arc fields per isocenter (one to two fields are used for the inferior isocenter). Isocenters are placed along the longitudinal axis, with no lateral or antero-posterior shifts. Isocenters are typically separated by no more than 24 cm. The collimator angle is at 90°C so that the MLC leaves move along the longitudinal direction of the patient. Asymmetric jaws are used along the patient’s longitudinal direction so that two arc fields at each isocenter are coplanar and cover different lengths of the patient body.

**Figure 1:**
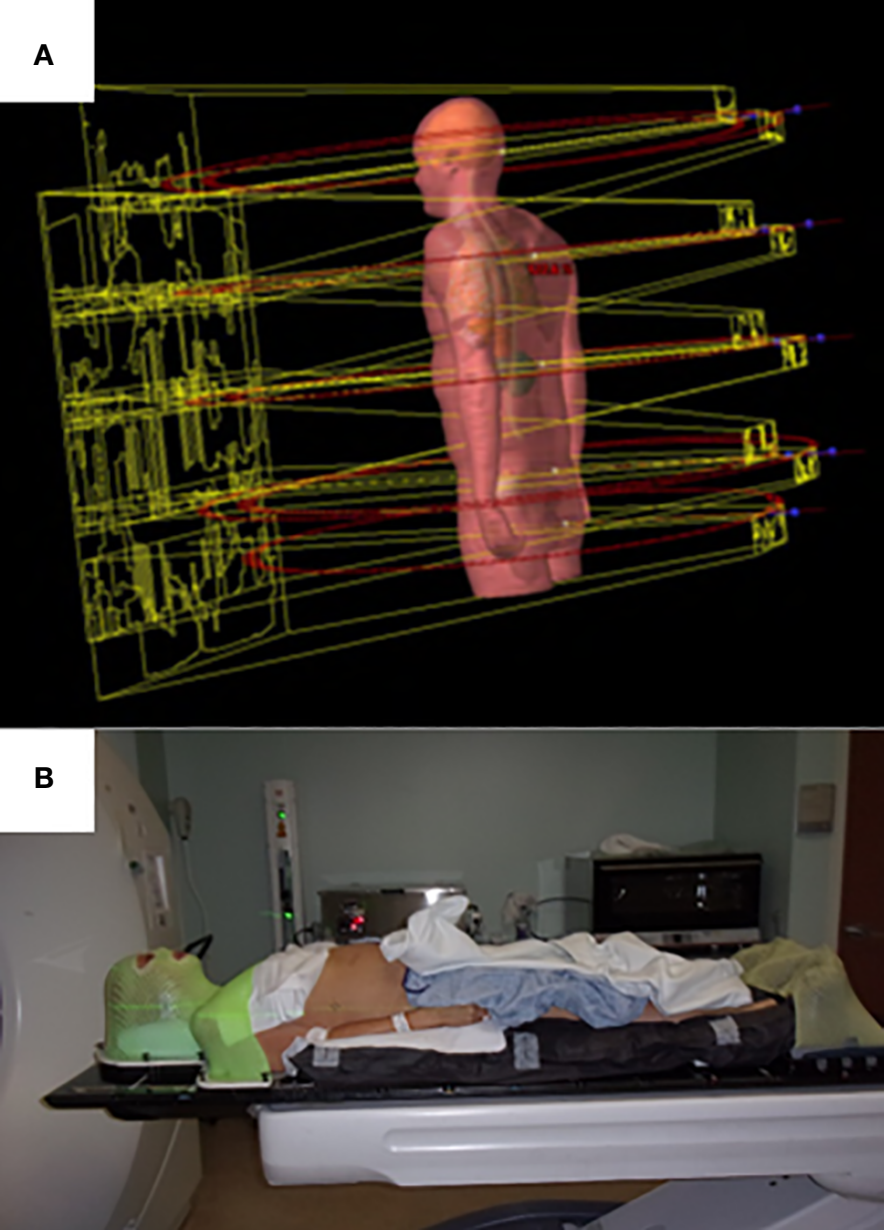
Upper body field arrangement **(A)** and setup **(B)** for patient treated with VMAT TMLI.

For the lower body, from the mid-thigh to the bottom of the feet, either a VMAT or three-dimensional technique can be used. For the VMAT technique, typically three large aperture, coplanar fields are used. For the three-dimensional technique, three to four static AP/PA photon fields in two to three isocenters are planned in a feet-first supine position. Plans are generated using a six-megavoltage photon beam for all VMAT fields and optimization for all isocenters is carried out simultaneously. The automatic feathering option for the optimizer was enabled in plan optimization, which leads to a smooth dose gradient with each VMAT field in the dose junction regions to minimize dose variation due to setup uncertainties. The upper body VMAT TMLI plan is summed with the lower extremity plans for verification of adequate dose in the junction region at the upper thigh. Visualization of field arrangement is shown in **Figure 1A**.

Treatments are planned to total doses of 12-20 Gy based on clinical protocol, given in 1.5-2 Gy fractions, respectively. For all plans dose was prescribed to skeletal bones (excluding the ribs and skull), ribs, skull, lymph nodes, spinal canal, and spleen. For the 20 Gy plans, the brain and liver are treated to 12 Gy. Plans are optimized such that a minimum of 85% of the PTV received prescription dose. Planning dose constraints include mean lung dose of less than 8 Gy based on prior toxicity analyses from our institution and association with survival in a Children’s Oncology Group study (18). This objective is achieved by prioritizing lung sparing over target coverage in the thoracic region. The maximum dose to normal organs was based off reference tables generated by historical TMLI plan data from our institution. Overall hotspots were limited as possible.

Plan optimization typically requires at least 2-3 hours. At our institution, we have developed a standalone application to automatically optimize VMAT TMLI plans. This application can be run overnight without user intervention. This application is written in the C# programming language and is built on top of the Eclipse Scripting Application Programming Interface (ESAPI) from Varian Medical Systems. The dosimetrist first delineates the structures and sets up the fields. Then this application is run to optimize the plan. All the VMAT fields at multiple isocenters are optimized in one single plan. The application automatically applies optimization parameters to the targets and normal organs; it also sets up other relevant parameters for the optimizer. The optimization parameters are dosimetric parameters used in construction of the objective function for optimization. These include upper and lower dose-volume objectives for each target, upper dose-volume objectives for normal organs, mean dose objectives for certain organs, and priority values for each objective. Other relevant parameters for the optimizer” refer to those settings that are not dosimetric constraints but are used by the optimizer. Examples of such settings are whether jaw tracking will be used in optimization and the MU objective in optimization. The optimization parameters are determined from prior dosimetric planning experience. At the end of optimization, the application calculates the plan dose and performs dosimetric evaluation. The application will re-optimize the plan automatically if necessary to improve the dosimetric quality of the treatment plan. The application checks plan quality by evaluating representative dosimetric parameters. If certain dosimetric parameters do not meet planning criteria, the application can adjust relevant optimization parameters and re-optimize the treatment plan. The in-house application currently does not change constraints and priority values based on individual patient anatomy. The application does not use artificial intelligence techniques.

#### 2.1.3 Treatment delivery

Prior to treatment delivery, plans undergo quality assurance with standard IMRT protocols. Currently our institution utilizes machine trajectory files and an independent calculation engine. A commercial software application (MobiusFX version 4.0, Varian Medical Systems, Inc., Palo Alto, California) analyzes trajectory log data after the VMAT TMLI fields are run on the Linac. The software calculates three-dimensional dose distribution based on the trajectory log data and compares the dose with the plan dose. Our institution uses three-dimensional Gamma analysis to check patient-specific QA results from the MobiusFX system. To facilitate more efficient treatment delivery, a separate setup appointment occurs either the Friday before or the day before the start of treatment to obtain imaging to aid with determining optimal patient alignment. Patients are immobilized using a thermoplastic mask in the head and neck and feet regions and a full body vacuum bag (**Figure 1B**). Treatments are then administered twice a day, with at least six hours between treatments. For image guidance, two cone beam (CB) CTs are obtained for each fraction: one in the head and neck isocenter region and one in abdominopelvic isocenter region. The CBCTs are registered to the simulation CT, with the shifts required for two CBCT scans averaged to correct the couch position. In addition, for each isocenter, orthogonal kilovoltage port films, at 45°C and 315°C in the thoracic region to obtain a clear view of the spine without obstruction from the arms, are taken to confirm accurate positioning. The 120 Gy plans were given in eight equal fractions with two fractions (separated by at least 6 hours) delivered each day. The 20 Gy plans were given in ten equal fractions with two fractions (separated by at least 6 hours) delivered each day.

### 2.2 Treatment plan analysis

**Figure 2:**
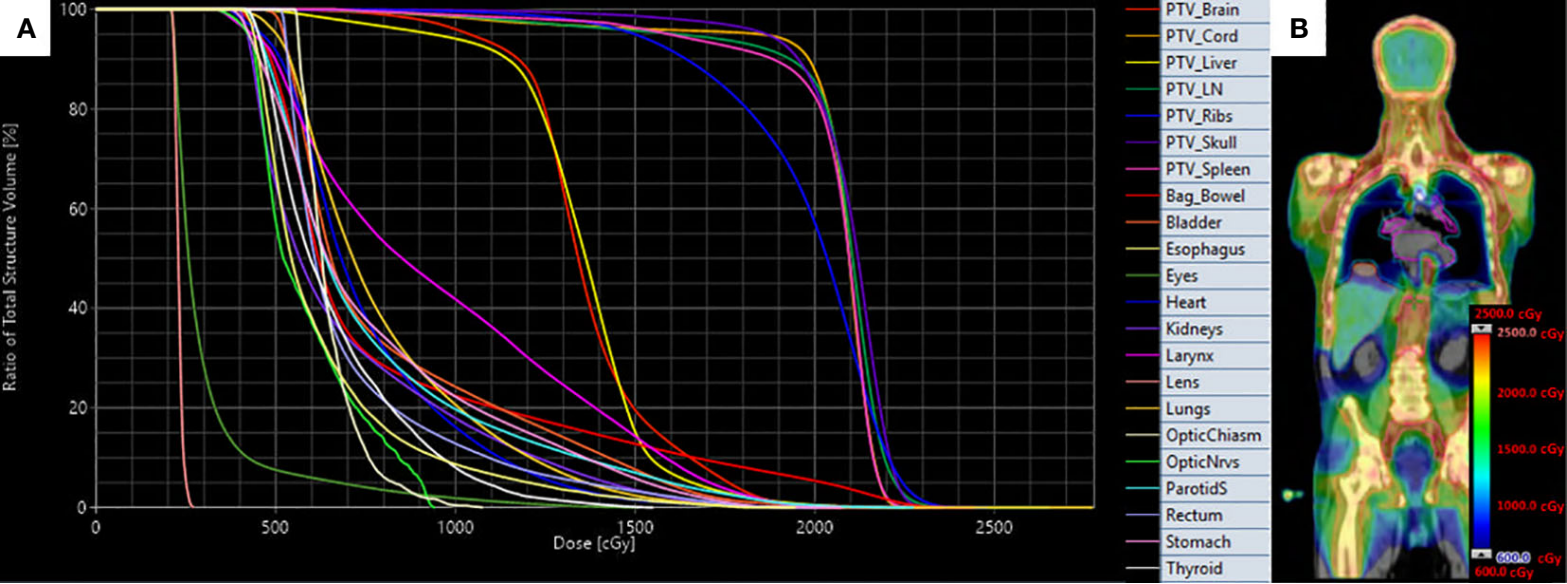
Target and organ-at-risk dose volume histograms **(A)** and three-dimensional dose distribution **(B)** of patient treated with VMAT TMLI to 20 Gy.

In this study, treatment plans were analyzed for the 20 Gy (10 patients) and 12-16 Gy (five patients) cohorts. An example treatment plan with dose-volume histograms (**Figure 2A**) and three-dimensional dose distribution (**Figure 2B**) of a patient treated to 20 Gy is visualized. We extracted the total duration of treatment for each fraction, calculated as the total time the patient was on the treatment table. Due to the first fraction requiring additional time, the first fraction was analyzed separate from remaining fractions. This time does include a brief break between treatment of the upper body and the legs where the patient is allowed to rest when orientation changes. We extracted and analyzed dosimetric parameters including mean and median dose (D50), D80, and D10 for all plans. The TMLI treatment plan for each patient was retrieved and the treatment plan data containing dose and structure contours were exported as DICOM files. In-house software applications were developed to extract and analyze dosimetric parameters for the targets and normal organs from the DICOM data files. To illustrate the spread of values for each dosimetric parameter, we calculated and presented the 1st quartiles and 3rd quartiles, in addition to the average values, for each dosimetric parameter in each cohort, where the 1st quartile is defined as the middle value between the minimum value and the median value, and the 3rd quartile is defined as the middle value between the maximum value and the median value for a given parameter. Statistical analysis in this study was performed with a data analysis software system (Excel version 2102, Microsoft Corp., Redmond, WA).

## 3 Results

A total of 15 patients’ treatment plans were analyzed. All patients were diagnosed with acute myeloid leukemia (AML). None patients were male. Median age was 53 (range 25-69). Median height was 167.5 cm (range 155-186 cm). Median weight was 70.2 kg (range 46.9-95.5 cm).

**Table 1** lists treatment duration statistics for the VMAT TMLI plans. The median treatment time for the first and subsequent fractions was 1.5 (range: 1.2-2.4) and 1.1 (range: 0.6-1.9) hours, respectively. The mean treatment time decreased from 1.3 hours for the first two patients treated in 2017 to 0.9 hours for the final two patients treated in 2022.

**Table 1:** Statistics of treatment times for patients treated with VMAT TMLI.

	First Fraction (hrs)	Subsequent Fractions (hrs)
Mean	1.6	1.1
Median	1.5	1.1
Range	1.2-2.4	0.6-1.9
Interquartile range (IQR)	1.3-1.9	1.0-1.2

**Table 2** lists mean dose and median dose statistics (average, standard deviation, and 1st and 3rd quartiles) for each structure with the 20-Gy cohort. Of note, the brain and liver were prescribed 12 Gy while the other target volumes were prescribed 20 Gy. All the target volumes had a mean dose greater than the prescribed dose except the ribs, which had an average mean dose of 19.5 Gy. The skeletal bones had an average mean dose of 21.1 Gy. The brain and liver have average mean doses of 14.8 and 14.1 Gy, respectively. Relative to the prescription dose of 20 Gy, the average mean dose for the normal organ volumes ranged from 16.5% to 72.0%, and the average median dose for the normal organs ranged from 16.5% to 71.0%. Among the normal organ structures, the lenses showed the lowest average mean and median dose values while the female breasts showed the highest average mean and median dose values. The mean lung dose had an average of 7.6 ± 0.6 Gy for the 20-Gy cohort. **Table 3** lists statistics of D80 and D10 for targets and normal organs with the 20-Gy cohort.

**Table 2:** Statistics of mean dose and median dose (D50) for each structure with the 20-Gy cohort.

	Mean dose (Gy)			Median dose (Gy)		
Structure	Avg ± StdDev	1st quartile	3rd quartile	Avg ± StdDev	1st quartile	3rd quartile
Skeletal Bones	21.1 ± 0.2	20.9	21.2	21.4 ± 0.4	21.2	21.6
Lymph Nodes	20.5 ± 0.5	20.3	20.8	21.3 ± 0.6	21.0	21.7
Spinal Canal	20.5 ± 0.5	20.3	20.6	21 ± 0.7	20.7	21.1
Skull	20.7 ± 0.7	20.1	21.2	21.2 ± 0.7	20.7	21.6
Ribs	19.5 ± 0.7	19.2	19.8	20.5 ± 0.8	20.2	20.8
Bladder	9.3 ± 1.5	8.3	10.2	8.4 ± 1.8	7.0	9.6
Body	13.3 ± 1.5	12.1	14.2	15 ± 1.6	14.1	16.0
Brain	14.8 ± 1.1	13.9	15.1	14.6 ± 1.2	13.7	14.8
Breasts	14.4 ± 0.8	14.0	14.8	14.2 ± 1.2	13.6	14.8
Esophagus	6.8 ± 1.4	6.1	7.0	6.1 ± 1.2	5.5	6.1
Eyes	4.2 ± 1	3.2	5.1	3.7 ± 0.9	3.0	4.3
Heart	7.5 ± 1.1	7.0	7.6	6.6 ± 1.3	6.1	6.8
Kidneys	7.5 ± 0.5	7.0	7.8	6.2 ± 0.5	5.7	6.6
Lens	3.3 ± 1	2.4	4.2	3.3 ± 1	2.4	4.1
Liver	14.1 ± 1.1	13.5	14.4	14.2 ± 1	13.5	14.9
Lower GI	9.6 ± 1.6	8.3	11.1	8.5 ± 2	7.0	10.4
Lungs	7.6 ± 0.6	7.4	7.9	6.7 ± 0.5	6.4	7.1
Oral Cavity	4.8 ± 1	4.0	5.3	3.9 ± 1	3.1	4.5
Ovaries	6.7 ± 3.1	4.9	7.9	6.1 ± 3	4.3	7.1
Parotids	8.2 ± 1	7.6	8.7	7.3 ± 1	6.6	7.7
Rectum	6.7 ± 0.9	6.1	7.2	5.7 ± 0.8	4.9	6.3
Thyroid	7.7 ± 1.2	6.8	8.1	7.1 ± 1.2	6.2	7.9
Upper GI	8.6 ± 1.3	7.8	9.1	7.7 ± 1.4	6.8	8.1

**Table 3:** Statistics of D80 and D10 for each structure with the 20-Gy cohort.

	D80 (Gy)			D10 (Gy)		
Structure	Avg ± StdDev	1st quartile	3rd quartile	Avg ± StdDev	1st quartile	3rd quartile
Skeletal Bones	20.4 ± 0.2	20.3	20.6	22.6 ± 0.6	22.2	22.9
Lymph Nodes	20.1 ± 0.4	19.7	20.3	22.5 ± 0.6	22.0	23.0
Spinal Canal	20.1 ± 0.4	20.0	20.2	21.8 ± 0.8	21.3	22.5
Skull	19.7 ± 0.6	19.5	20.1	22.6 ± 1	22.1	23.0
Ribs	17.3 ± 1.5	16.0	18.1	22.5 ± 0.7	22.1	23.0
Bladder	6.4 ± 1.2	5.7	7.3	14.4 ± 2	13.4	15.3
Body	5.1 ± 3.4	3.2	7.1	21.7 ± 0.5	21.4	22.1
Brain	13.2 ± 0.8	12.5	13.6	17.6 ± 1.5	16.8	17.9
Breasts	11.2 ± 0.5	10.9	11.5	19.3 ± 1	18.8	19.7
Esophagus	5.1 ± 0.9	4.8	5.2	10 ± 2.5	8.6	10.1
Eyes	3 ± 0.8	2.4	3.4	6.4 ± 2.1	4.3	7.3
Heart	5.1 ± 1.1	4.5	5.6	11.7 ± 1.6	11.2	12.3
Kidneys	4.9 ± 0.5	4.6	5.2	12.6 ± 1	12.0	12.8
Lens	2.8 ± 0.8	2.2	3.1	4 ± 1.6	2.5	5.5
Liver	12.8 ± 0.7	12.4	13.4	16.7 ± 1.9	15.5	17.0
Lower GI	6.2 ± 1.5	5.0	7.9	16 ± 2.1	15.4	17.3
Lungs	5.4 ± 0.4	5.1	5.8	11.5 ± 1.3	11.5	12.1
Oral Cavity	3 ± 0.7	2.4	3.4	8.6 ± 2	7.5	9.7
Ovaries	4.7 ± 1.3	4.0	5.2	9.4 ± 5.2	6.5	11.5
Parotids	5.5 ± 0.9	5.2	5.8	12.9 ± 1.6	11.5	13.9
Rectum	5.1 ± 0.7	4.5	5.7	10.3 ± 2.2	9.5	11.1
Thyroid	5.6 ± 0.9	5.0	5.8	11.3 ± 1.7	9.8	12.7
Upper GI	6.2 ± 1.1	5.4	6.4	12.9 ± 2.2	11.3	13.5

**Figure 3:**
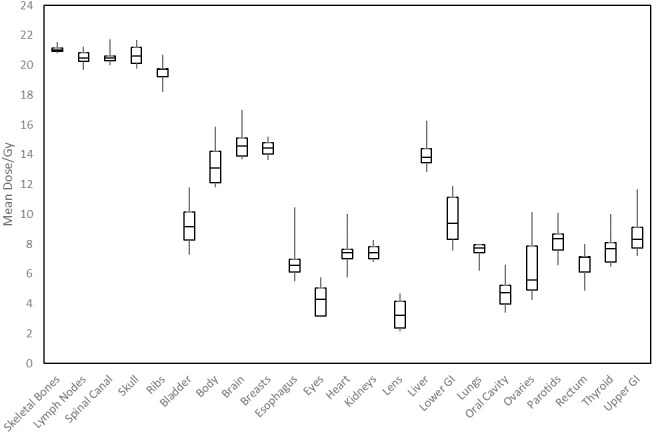
Distribution of mean dose (D_mean_) for target volumes and major normal organs in the 20-Gy cohort. The median value of D_mean_ for each structure is shown at the horizontal bar in the middle of each rectangle. The 1st and 3^rd^ quartiles are shown as the lower and upper horizontal sides of each rectangle. The minimum and maximum range of D_mean_ is shown as the vertical lines extending from each rectangle.

**Figure 4:**
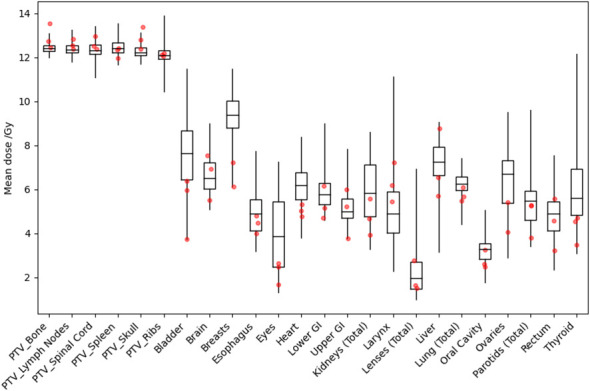
Distribution of mean dose (D_mean_) for target volumes and major normal organs in the historical 12-Gy TMLI treatment plans. The median value of D_mean_ for each structure is shown at the horizontal bar in the middle of each rectangle. The 1st and 3rd quartiles are shown as the lower and upper horizontal sides of each rectangle. The minimum and maximum range of D_mean_ is shown as the vertical lines extending from each rectangle. The red dots are D_mean_ data in the three VMAT TMLI treatment plans in the 12-Gy cohort.

**Figure 3** shows distributions of mean dose for target volumes and major normal organ volumes in the VMAT TMLI plans for the 20-Gy cohort. The minimum, maximum, and first, second, and third quartiles of the mean dose are shown in the box plot for each target and each normal organ. **Figure 4** shows the mean dose data to targets and normal organ volumes in the three 12-Gy VMAT TMLI treatment plans overlain over dosimetry from our historic 12-Gy helical tomotherapy cohort. Additional dose statistics for the two patients treated with 14 or 16 Gy are shown in **Table 4**.

**Table 4:** Dose statistics for patients treated to 14 and 16 Gy.

	14 Gy				16 Gy			
Structure	Mean dose (Gy)	Median dose (Gy)	D80 (Gy)	D10 (Gy)	Mean dose (Gy)	Median dose (Gy)	D80 (Gy)	D10 (Gy)
Skeletal Bones	14.86	15.04	14.43	15.79	16.65	16.98	16.3	17.81
Lymph Nodes	14.49	15.2	14.43	15.89	16.21	16.96	16.21	17.77
Spinal Canal	14.84	14.77	14.46	15.52	16.25	16.73	15.6	17.26
Skull	14.89	15.27	14.21	16.2	16.3	16.91	16.14	17.82
Ribs	14.13	14.9	13.47	15.84	16.3	16.72	15.63	17.68
Bladder	5.43	4.57	3.98	8.6	7.91	7.97	5.68	10.68
Body	8.07	8.67	0.63	15.06	11.24	12.56	5.67	17.29
Brain	5.73	4.33	2.7	11.99	9.03	8.49	4.38	15.24
Breasts	8.39	7.96	6.19	12.03	N/A	N/A	N/A	N/A
Esophagus	4.43	3.84	3.42	6.31	5.28	4.39	3.88	8.11
Eyes	3.06	2.67	2.37	4.58	2.64	2.55	2.2	3.28
Heart	4.27	3.94	2.86	6.26	6.32	5.47	4.06	10.25
Kidneys	4.33	3.65	3.21	6.65	5.85	5.09	3.99	9.14
Lens	2.39	2.4	2.19	2.68	2.27	2.24	2.11	2.47
Liver	7.56	6.22	5.08	12.83	10.27	9.69	7.73	14.75
Lower GI	5.91	5.1	3.64	9.83	8.27	8	5.16	12.75
Lungs	5.98	5.28	4.16	9.51	6.99	5.9	4.66	11.32
Oral Cavity	3.18	2.49	1.95	6.14	3.41	3.02	2.07	5.62
Ovaries	4.53	4.05	3.68	5.81	N/A	N/A	N/A	N/A
Parotids	4.57	4.06	3.34	6.72	6.02	5.7	4.07	9.01
Rectum	4.72	3.98	3.48	7.75	5.47	4.9	4.36	7.56
Thyroid	4.72	4.34	3.9	6.27	5.76	5.3	4.55	7.94
Upper GI	4.45	3.79	3.33	6.93	6.91	6.48	5.46	9.38

N/A, not applicable.

## 4 Discussion

This study details the treatment technique our institution has used for our initial fifteen VMAT based TMLI patients, as well as a dosimetric summary of the resulting treatment plans. Using this technique, suitable target volume coverage and normal organ sparing is achievable, even with dose escalation to 20 Gy. Our approach could be used by other institutions to implement TMLI using VMAT, particularly when helical tomotherapy is not available.

Han et al. have performed the largest dosimetric analysis to date of patients treated with TMLI, including patients treated to an escalated dose of 20 Gy (17). Their analysis included a total of 120 patients treated to 20 Gy, with almost all patients treated with helical tomotherapy (four patients were treated with VMAT). The dosimetry in our study, with average mean dose to the target volumes ranging from 19.5 Gy (ribs) to 21.1 Gy (skeletal bones) is nearly identical to their cohort, where doses ranged from 19.3 Gy (ribs) to 20.8 Gy (skeletal bones). Dosimetry to the intermediate dose regions of the brain and liver were also similar, at 14.8 and 14.1 Gy, respectively, in our study and 13.6 and 12.9 Gy, respectively, in their study. Normal organ dose ranged from 13.0-76.0% in their study compared to 16.5% to 72.0% in our study, corresponding to lens dose and female breast dose in both studies. The same trends can be applied to the three patients treated to 12 Gy in our study. Although we had insufficient numbers to generate descriptive statistics, the mean doses fell within the same range as our historical helical tomotherapy cohort. In total, these data support the idea that VMAT TMLI can achieve comparable dosimetry to plans generated using helical tomotherapy. The ability to deliver TMLI with VMAT is critical for more widespread availability of TMLI as a treatment option when designing transplant conditioning regimens. Initial trials of TMLI utilized helical tomotherapy due to increased ease of treating lengths of the body without requiring several isocenters (10–13). However, traditional C-arm linear accelerators are more commonly available in radiation oncology departments, so VMAT would facilitate implementation of TMLI in institutions where it otherwise would not be feasible.

The feasibility of VMAT TMI was first established in 2011 (19, 20). VMAT was shown to lead to comparable dosimetry to helical tomotherapy approaches, as well as a reduction in beam on time. However, it is important to note that treatment time is still significant due to additional time required to set up multiple fields and isocenters, as shown in **Table 1**, though the total treatment time is still comparable to our experience with helical tomotherapy, where the median treatment time for the first and subsequent fractions was 1.6 (range: 1.2-2.6) and 1.4 (range: 0.6-2.3) hours, respectively. Further improvements in workflows therefore may be able to better capitalize on VMAT to speed up treatment delivery. Importantly, using flattening filter free beams would not decrease treatment time, as it is maximum gantry speed and not dose rate that is the primary limitation on delivery time.

Compared to helical tomotherapy, VMAT has the potential advantage of allowing dose rate modulation in certain anatomical regions, although the dose rate effect on normal tissue complications needs further investigation. Most modern C-arm linacs allows the user to change the nominal dose rate for each VMAT field. Currently, the maximum nominal dose rate of 600 monitor units (MU)/min was used in all the VMAT TMLI fields at our institution. At this nominal dose rate, effective dose rates between VMAT and helical tomotherapy techniques are comparable: with a fractional dose of 2 Gy, the dose rate to targets and lung are 1.6 Gy/min and 0.8 Gy/min, respectively, with VMAT compared to 1.8 Gy/min and 0.9 Gy/min with helical tomotherapy. If the nominal dose rate for VMAT fields in the lung region is reduced by 100 MU/min, the effective dose rate to the lung can be further reduced at the expense of longer beam-on time in the lung region.

Several subsequent studies have now been performed using VMAT TMLI. An initial report by Han et al. was the first to compare VMAT TMLI plans to helical tomotherapy plans (21). In this analysis, VMAT plans were found to have a more than 10% reduction of average median dose in 16 organs. Further, beam-on time for VMAT plans was about 50% shorter. Larger studies have evaluated the use of VMAT TMLI to a total dose of 12 Gy (22, 23). In the study by Mancosu et al., plans for 21 patients were evaluated, demonstrating 95% of prescription dose covered greater than 99% of the PTV in junctional regions between isocenters (22). In the study by Loginova et al, 157 patients treated with helical tomotherapy and 52 patients treated with VMAT were evaluated (23). There were no observed differences in acute, subacute, or late toxicities, as well as similar dose distributions. In total, these studies in combination with the present study demonstrate the overall feasibility and desirability of implementation of VMAT TMLI techniques.

This study is limited due to sample size, particularly for treatment doses less than 20 Gy. However, the dosimetry is consistent with prior reports, and to our knowledge this is the largest dosimetric report of dose-escalated VMAT TMLI to date. Due to a lack of dose escalated VMAT TMLI in the literature, this study further supports its implementation, at both standard myeloablative doses and escalated doses. Though implementation of VMAT TMLI is logistically challenging and is still associated with long treatment times due to requiring multiple image acquisitions for patient setup, we are actively seeking to further optimize planning and clinical workflows, which will be crucial for making TMLI more widely available in the radiation oncology community.

## 5 Conclusions

Treatment delivery with VMAT has the potential to increase availability of TMLI. Based on our initial experience, delivery of TMLI with VMAT is feasible and achieves target volume and normal organ dosimetry comparable to historical helical tomotherapy TMLI plans. Treatment procedures detailed in this study, and resulting dosimetry, can be used to inform implementation of VMAT TMLI at other institutions.

## Data availability statement

The raw data supporting the conclusions of this article will be made available by the authors, without undue reservation.

## Ethics statement

Ethical review and approval was not required for the study on human participants in accordance with the local legislation and institutional requirements. Written informed consent for participation was not required for this study in accordance with the national legislation and the institutional requirements.

## Author contributions

CL performed data collection, data analysis, and manuscript preparation. CH participated in the design of this study and performed data collection and data analysis. AL participated in the design of this study and performed critical review of the manuscript. JW is recipient of funding from Varian Inc. to support the development, treatment planning and implementation of TrueBeam based TBI and TMLI at this center. He participated in the design of this study, creation of methods for this study, and data analysis, and performed critical review of the manuscript. All authors contributed to the article and approved the submitted version.
